# Relationships between patient activation, disease-specific knowledge and health outcomes among people with diabetes; a survey study

**DOI:** 10.1186/1472-6963-14-393

**Published:** 2014-09-16

**Authors:** Michelle Hendriks, Jany Rademakers

**Affiliations:** NIVEL- Netherlands institute for health services research, PO Box 1568, 3500 BN Utrecht, the Netherlands

**Keywords:** Patient activation, Diabetes mellitus, Patient characteristics, Health-related outcomes

## Abstract

**Background:**

Nowadays, patients are expected to be involved in their health care, well-informed and able to adjust their behavior to maintain a good health. Investigating patient activation and its relationships with patient characteristics and health-related outcomes will provide further insight into the gains to be expected if patients are more involved in their healthcare.

**Methods:**

Based on claims data, 5,346 people were selected who received diabetes care in the last 12 months. They received a questionnaire including the Patient Activation Measure (PAM) and questions on patient characteristics, life style and self-management behaviors, knowledge about diabetes, healthcare utilization and own clinical values. We used bivariate analyses and regression analyses to investigate the relationships between patient characteristics, patient activation level, and health-related outcomes.

**Results:**

Data of 1,845 (35%) people were used in the analyses. Patient activation differed depending upon several patient characteristics. Patient activation level was positively related to getting the recommended feet and eye examinations after controlling for several patient characteristics; no association was found for life-style and self-management behaviors and the other healthcare utilization measures. Those with a low patient activation level less often reported to have knowledge about diabetes and of their values on clinical indicators.

**Conclusions:**

Among people with diabetes, patient activation level was especially related to disease-specific knowledge and less with health-related behavior and outcomes. The PAM might therefore especially be an useful instrument for clinical practice to identify patients most in need for diabetes education.

## Background

Patients and especially the chronically ill are more and more expected to be in charge of their own health and the healthcare they receive. For one, several western countries have introduced some form of managed competition in their healthcare system with the aim to accomplish a more efficient and more patient-centered healthcare system. Patients are expected to take more responsibility, to inform themselves about existing treatments and differences in quality of care between healthcare providers, and to choose the treatment and provider that best meet their needs.

Also, the Chronic Care Model has been introduced to help healthcare providers to improve patients’ health outcomes [1, 2]. One important element is to accomplish productive interactions that help patients to set goals and solve problems for improved self-management. Healthcare professionals should mainly act as coaches, providing patients with information and helping them to develop skills to take a leading role [3, 4]. Both these trends ask for active patients; patients who are involved in their own health care, well-informed and able to adjust their behavior to realize or maintain a good health. For the chronically ill, it additionally means employing self-management activities such as monitoring one’s condition, self-treatment and coordinating the care of different providers [5].

Diabetes is one of the most prevailing chronic conditions and a major cause of morbidity, disability and mortality [6]. Self-management has been recognized as an important contributor to improved health outcomes for people with diabetes [7]. However, not all people are equally well equipped to perform the variety of activities required for proper self-management. Educating and training people to fulfill the role of active patient is assumed to have positive effects on health outcomes. It will also most likely enhance the sustainability of healthcare systems. More activated patients will be better able to self-manage their disease leading to a lower uptake of more costly health care [7–9].

In order to enhance the activation level of people with diabetes or a chronic illness in general, an instrument is needed to assess their abilities to manage their disease. Hibbard and colleagues developed the Patient Activation Measure (PAM) for this purpose. They define patient activation as someone’s knowledge, skills, confidence and behaviors needed for self-managing one’s condition or health [9, 10]. Research suggests that people go through four stages of patient activation. At stage 1, people tend to be overwhelmed and unprepared to play an active role, they are predisposed to be passive recipients of care. At stage 2, individuals lack knowledge and confidence for self-management. At stage 3, people are beginning to take action but may still lack confidence and skills to support new behaviors. Finally, at stage 4, people have confidence and perform adequate behaviors but may not be able to maintain them in the face of stress.

Patients with a higher activation level are more likely to engage in healthy behaviors such as regular exercise and proper diet, to engage in disease-specific self-management behaviors, to report medication adherence and to obtain preventive care [8, 11, 12]. Higher patient activation also appears to be related with better biometrics such as blood pressure and lipoprotein levels within a normal range and with less hospitalizations and emergency department visits [8]. Several studies have shown that care tailored to a patient’s activation level as measured with the PAM resulted in improved values on clinical indicators, higher adherence to medication regimens and a reduction in hospitalizations and emergency department visits [8, 13]. Also, patient activation appeared to be modifiable and increases in activation were followed by improvement in self-management behaviors [13, 14].

Up till now, only a few studies focused on the activation level of people with diabetes. These studies have shown that patient activation is related to age, gender, race/ethnicity, self-reported health, duration and severity of the disease and comorbidity [6, 7, 15]. Patient activation was also related to life-style and self-management behaviors and healthcare utilization of people with diabetes. Rask et al., for instance, found that more active patients reported higher rates of weekly feet checks, getting recommended eye examinations, regular exercise and ease of managing diabetes [15]. An Australian study revealed that individuals with a lower patient activation level were more likely to have been hospitalized for diabetes-related complaints or to have visited the emergency departments [7]. So far, there has been one longitudinal study showing that a high activation level was predictive of better rates for hemoglobin A1C (HbA1C) checkups, low-density lipoprotein cholesterol (LDL-C) checkups, HbA1C control and all-cause inpatient discharges [6]. Patient activation did not predict lipid-lowering drug use, LDL-C control and hospital discharges with a primary diagnose of acute myocardial infarction.

In short, previous studies among diabetes patients have shown that patient activation as measured with the PAM is related to patient characteristics and to health-related outcomes. However, studies with the PAM are scarce, especially outside the USA. Investigating patient activation and its relationships with health-related outcomes in different patient groups and countries will provide further insight into the gains to be expected if patients are more involved in their healthcare. In this study, we focused on people with diabetes in the Netherlands and determined the relationships between patient characteristics, health-related outcomes and patient activation. We will answer the following questions: (1) What is the activation level of Dutch people with diabetes; and (2) How does patient activation relate to patient characteristics and self-reported health-related outcomes in this group?

## Methods

### Subjects and design

As part of a larger study on patients’ experiences with diabetes care, 23,074 people who received diabetes care in 2010 were selected from claims data of six health insurance companies. The health insurance companies selected individuals who claimed costs for diabetes care provided in 61 selected diabetes care networks from April to December 2010. Next, people younger than 18 years and/or who were approached in the past 12 months for other surveys on patient experiences were excluded. Each health insurance company randomly selected 300 patients per diabetes care network. If less than 300 patients received care from one of the networks all patients were selected with a minimum of 80 patients per network. This resulted in the total of 23,074 people.

The individuals received a paper questionnaire at home in the beginning of 2011 and non-respondents received up to three reminders (Dillman method) [16]. The questionnaire included questions on patient characteristics, experience with diabetes care and health-related outcomes. A subgroup of 5,346 people received a questionnaire that also contained the PAM. Ethical approval of the study was not necessary as research by means of surveys that are not taxing or hazardous for patients (i.e. the once-only completion of a questionnaire containing questions that do not constitute a serious encroachment on the respondent) is not subject to the Dutch Medical Research Involving Human Subjects Act (WMO).

### Patient characteristics

The following demographic data were collected: (1) gender; (2) age in eight categories reclassified as 18–54 years, 55–64 years, 65–74 years, 75 years and older; (3) education level in eight categories reclassified as low (no education through lower vocational education), middle (intermediate secondary through higher secondary education) and high (higher vocational education or university); (4) ethnicity (Dutch, Western, or non-Western); (5) spoken language at home (Dutch or other); (6) disease duration, that is time since the diagnosis of diabetes was established (less than 12 months, 1 to 2 years, 2 to 10 years or more than 10 years); and (7) self-reported health status (poor, fair, good, very good or excellent).

### Health-related outcomes

#### Life style and self-management behaviors

Respondents were asked whether they smoke (no or yes) and whether they check their own blood glucose level (no or yes).

#### Self-perceived knowledge about diabetes

The questionnaire had three questions concerning knowledge about diabetes: whether the person knows what type of diabetes he/she has (no or yes), knows which side-effects of medications he/she should attend to (no, not really, on the whole yes or yes) and knows which actions to take when his/her blood glucose level is too high (no, not really, on the whole yes or yes).

#### Healthcare utilization

The use of routine checkups was assessed with five questions on how long ago the following checkups had taken place: blood sample to check HbA1C level, blood sample to check cholesterol level, blood pressure measurement by healthcare provider, feet check by doctor and eye examination. Dutch guidelines for diabetes care prescribe that all checkups except the eye examination take place at least once every year; eye examinations have to take place at least once every two years. Following these guidelines, we used the following answer categories for all checkups except the eye examination: within the past 12 months (reflecting proper diabetes care), over 12 months ago or never, or don’t know. For eye examination the following categories were used: within the past 24 months (reflecting proper diabetes care), over 24 months ago or never, or don’t know.

#### Self-reported values on clinical indicators

Respondents were asked to indicate their HbA1C value (between 2-7% (0–53 mmol/mol), between 7-9% (53–75 mmol/mol), between 9-24% (75–240 mmol/mol) or don’t know), diastolic blood pressure (lower than 90, 90 or higher or don’t know) and systolic blood pressure (lower than 140, 140 or higher or don’t know) as measured during the last measurement.

### Patient Activation Measure (PAM)

The PAM 13-Dutch consists of 13 items assessing knowledge, skill and confidence for self-care [17]; see Table 1 for the original American version. The PAM has recently been translated in Dutch and validated in a sample of chronically-ill people including people with diabetes and a sample of the general population [17, 18]. All items have five possible responses with scores ranging from 0 to 4; (1) *disagree strongly*, (2) *disagree*, (3) *agree*, (4) *agree strongly* or (0) *not applicable*. Internal consistency of the PAM in this study was good (Cronbach’s alpha = 0.86).

**Table 1:** **Original American version of 13 item Patient Activation Measure**

1	When all is said and done, I am the person who is responsible for managing my health condition
2	Taking an active role in my own healthcare is the most important factor in determining my health and ability to function
3	I am confident that I can take actions that will help prevent or minimize some symptoms or problems associated with my health condition
4	I know what each of my prescribed medications do
5	I am confident that I can tell when I need to go get medical care and when I can handle a health problem myself
6	I am confident I can tell my healthcare provider concerns I have even when he or she does not ask
7	I am confident that I can follow through on medical treatments I need to do at home
8	I understand the nature and causes of my health condition(s)
9	I know the different medical treatment options available for my health condition
10	I have been able to maintain the lifestyle changes for my health that I have made
11	I know how to prevent further problems with my health condition
12	I am confident I can figure out solutions when new situations or problems arise with my health condition
13	I am confident that I can maintain lifestyle changes like diet and exercise even during times of stress

For calculating patients’ PAM score, we followed the guidelines of Insignia Health [19]. Participants who filled out less than seven questions or answered all items with *disagree strongly* or *agree strongly* were excluded. We calculated the mean score on the PAM items leaving out items deemed not applicable by the respondents. This mean score was transformed into a standardized activation score ranging from 0 to 100, the PAM score. Next, the PAM score was converted into the four levels of patient activation conform the scoring rules of Insignia Health [19].

### Statistical analyses

Bivariate analyses were conducted to investigate the relationship between patient characteristics, health-related outcomes and the PAM score. A two-sample *t*-test was used to test for significant differences in PAM score for binary variables and one-way ANOVA was used for categorical variables with three or more groups.

Using Stata 13.1, we performed regression analyses to evaluate the relationship between the PAM level (predictor variable with PAM level 1 as reference group) and each of the health-related outcomes (dependent variables) while controlling for patient characteristics. Logistic regression analyses were used for the binary health-related outcomes, that is smoking status, self-checking blood glucose level and knowledge of type of diabetes. For the health-related outcomes with three or more groups, we performed ordered logistic regression analyses in case the response categories were ordered (i.e., knowledge about side-effects of medicines and knowledge about what to do when blood glucose level is too high) and multinomial logistic regression analyses in case the response categories had no natural ordering (i.e. outcomes concerning healthcare utilization and self-reported clinical values).

## Results

### Respondents

The response of the total study was 8,609 (response rate 37%). The group of respondents consisted of significantly more men than the group of non-respondents (48% versus 44%). Also, mean age was significantly lower in the group of respondents, but the difference with the group of non-respondents was less than 2.5 months.

1,969 of the 5,346 patients filled out the PAM. A total of 1,845 (response rate 35%) questionnaires were used for statistical analyses. 124 participants (6.3%) were excluded from the analyses; 72 participants because they answered less than seven of the PAM items and 52 participants since they responded to all items identically.

About half of the respondents were male (see Table 2). Most respondents were 55 years or older, had a low to middle education level, were of Dutch origin and spoke Dutch at home. For most respondents the diagnosis of diabetes was known for two years or longer and the large majority rated their general health as fair to good. The average PAM score of the respondents was 57.4 (SD = 14.3; see Table 2), which is relatively low compared to other studies [5, 7, 15, 17, 20]. About 23% of the respondents tended to be overwhelmed and unprepared to play an active role in their health care (PAM level 1). 23% of the respondents reported to lack knowledge and confidence for self-management (PAM level 2); 31% of the respondents were beginning to take action but may still lack confidence and skills to support new behaviors (PAM level 3) and 24% of the respondents reported to have confidence and to be able to perform adequate behaviors in most circumstances (PAM level 4).

**Table 2:** **Bivariate relations between patient characteristics and the Patient Activation Measure (PAM**)

	N	%	Mean PAM (SD)	p-value
Total	1,845		57.4 (14.3)	
Gender	1,810			**0.017**
Male	856	47.3	58.1 (14.3)	
Female	954	52.7	56.5 (14.2)	
Age	1,820			**<0.001**
18-54 years	305	16.8	59.6 (14.1)	
55-64 years	512	28.1	57.8 (14.3)	
65-74 years	573	31.5	57.3 (14.3)	
75 years and older	430	23.6	55.2 (14.0)	
Education level	1,732			**<0.001**
Low	997	57.6	56.5 (14.2)	
Middle	634	36.6	58.0 (13.8)	
High	101	5.8	62.7 (15.6)	
Ethnicity	1,820			0.555
Dutch	1,454	79.9	57.5 (14.5)	
Western	133	7.3	56.2 (14.0)	
Non-Western	233	12.8	57.0 (13.4)	
Spoken language at home	1,753			**0.008**
Dutch	1,617	92.2	57.6 (14.5)	
Other	136	7.8	54.2 (11.8)	
Disease duration	1,711			0.352
Less than 12 months	156	9.1	56.2 (14.3)	
1 year up to 2 years	154	9.0	58.9 (14.9)	
2 years up to 10 years	906	53.0	57.2 (14.0)	
More than 10 years	495	28.9	57.7 (14.7)	
General health status	1,817			**<0.001**
Poor	70	3.9	47.3 (13.5)	
Fair	577	31.8	53.1 (13.0)	
Good	1,014	55.8	59.1 (13.9)	
Very good	117	6.4	65.0 (14.6)	
Excellent	39	2.1	69.8 (15.7)	

Note. Bold values indicate a p-value < 0.05.

### PAM and patient characteristics

Table 2 also shows the relationships between the PAM score and patient characteristics. All patient characteristics except ethnicity and disease duration were significantly related to patient activation. More specifically, men, younger people and higher educated people scored higher on patient activation. The better someone’s self-reported general health, the higher the PAM score. Respondents who speak Dutch at home reported higher PAM scores than those who speak a foreign language.

### PAM and health-related outcomes

The bivariate analyses show that the PAM score was related to most health-related outcomes in the expected direction (see Table 3). We did not find significant differences in patient activation depending on smoking status, self-checking of blood glucose level, time since last blood sample taken to check HbA1C and time since last blood pressure measurement by a healthcare provider.

**Table 3:** **Bivariate relations between the Patient Activation Measure (PAM) and health-related outcomes**

	N	%	Mean PAM (SD)	p-value
**Life style and self-management**				
Smoking status	1,818			0.862
No	1,468	80.7	57.4 (14.4)	
Yes	350	19.3	57.5 (13.9)	
Self-checking blood glucose level	1,820			0.822
No	1,090	59.9	57.5 (14.7)	
Yes	730	40.1	57.3 (13.8)	
**Self-perceived knowledge**				
Knowledge of type of diabetes	1,781			**<0.001**
No	352	19.8	55.2 (14.6)	
Yes	1,429	80.2	58.1 (14.2)	
Knowledge about side-effects of medicines	1,317			**<0.001**
No	118	9.0	51.5 (12.8)	
Not really	282	21.4	52.6 (12.7)	
On the whole, yes	226	17.2	56.6 (13.0)	
Yes	691	52.5	60.6 (14.5)	
Knowledge about what to do when blood glucose level is too high	1,780			**<0.001**
No	285	16.0	54.8 (14.8)	
Not really	371	20.8	54.1 (13.5)	
On the whole, yes	249	14.0	56.5 (12.5)	
Yes	875	49.2	59.9 (14.5)	
**Healthcare utilization**				
Blood sample to check HbA1C	1,806			0.218
Within the past 12 months	1,672	92.6	57.5 (14.3)	
Over 12 months ago/Never	50	2.8	59.5 (16.1)	
Don’t know	84	4.7	55.2 (13.6)	
Blood sample to check cholesterol level	1,811			**0.007**
Within the past 12 months	1,598	88.2	57.7 (14.3)	
Over 12 months ago/Never	130	7.2	56.5 (13.2)	
Don’t know	83	4.6	52.8 (14.2)	
Blood pressure measurement by healthcare provider	1,821			0.132
Within the past 12 months	1,769	97.1	57.4 (14.3)	
Over 12 months ago/Never	40	2.2	58.4 (16.3)	
Don’t know	12	0.7	49.3 (10.6)	
Feet check by doctor	1,808			**0.004**
Within the past 12 months	1,428	79.0	57.9 (14.3)	
Over 12 months ago/Never	339	18.8	55.5 (14.2)	
Don’t know	41	2.3	53.3 (14.4)	
Eye examination	1,828			**0.001**
Within the past 24 months	1,698	92.9	57.7 (14.4)	
Over 24 months ago/Never	113	6.2	53.8 (13.1)	
Don’t know	17	0.9	48.9 (12.8)	
**Self-reported clinical values**				
Level of blood glucose	1,783			**<0.001**
Between 2% and 7%	717	40.2	59.2 (14.4)	
Between 7% and 9%	514	28.8	58.3 (14.1)	
Between 9% and 24%	72	4.0	54.9 (14.3)	
Don’t know	480	26.9	54.4 (13.6)	
Diastolic blood pressure	1,764			**<0.001**
Lower than 90	1,227	69.6	58.6 (14.2)	
90 or higher	232	13.2	56.3 (14.3)	
Don’t know	305	17.3	53.7 (13.1)	
Systolic blood pressure	1,788			**<0.001**
Lower than 140	924	51.7	58.8 (14.4)	
140 or higher	600	33.6	56.7 (13.9)	
Don’t know	264	14.8	53.9 (13.3)	

Note. Bold values indicate a p-value < 0.05.

The results of the regression analyses are presented in Table 4. After controlling for patient characteristics, patient activation level was significantly related to self-perceived knowledge about diabetes, that is: knowledge about type of diabetes, possible side-effects of medication to attend to, and actions to take when one’s blood glucose level is too high. In all instances, respondents in PAM level 1 reported less knowledge about diabetes compared to respondents in PAM level 2 to 4. No associations with activation level were found for smoking status or self-checking the blood glucose level. Concerning healthcare utilization, activation level was only related with time since last feet and eye examination. Respondents in PAM level 1 more often indicated that they did not have a feet examination in the last 12 months than respondents in PAM level 2 and 4, and they more often indicated that they did not have an eye examination in the last 24 months than respondents in PAM level 4. Finally, respondents in PAM level 1 more often lacked knowledge of their HbA1C value and blood pressure levels than respondents in PAM level 2 to 4.

**Table 4:** **Relationship of health-related outcomes to the Patient Activation Measure (PAM)**

	Coefficient PAM (ref: level 1)	SE	p-value
**Life-style and self-management**			
Smoking status (ref: yes)			
PAM level 2	-0.29	0.21	0.158
PAM level 3	0.17	0.19	0.351
PAM level 4	-0.12	0.21	0.559
Self-checking blood glucose level (ref: no)			
PAM level 2	0.20	0.17	0.225
PAM level 3	0.30	0.16	0.059
PAM level 4	0.27	0.17	0.113
**Self-perceived knowledge**			
Knowledge of type of diabetes (ref: no)			
PAM level 2	0.78	0.22	**<0.001**
PAM level 3	0.50	0.19	**0.010**
PAM level 4	0.64	0.22	**0.004**
Knowledge about side-effects of medicines			
PAM level 2	0.66	0.17	**<0.001**
PAM level 3	0.85	0.16	**<0.001**
PAM level 4	1.20	0.18	**<0.001**
Knowledge about what to do when blood glucose level is too high			
PAM level 2	0.33	0.14	**0.022**
PAM level 3	0.62	0.14	**<0.001**
PAM level 4	0.83	0.15	**<0.001**
**Healthcare utilization**			
Blood sample to check HbA1C over 12 months ago or never (ref: within the past 12 months)			
PAM level 2	-0.20	0.51	0.697
PAM level 3	0.09	0.45	0.835
PAM level 4	0.11	0.50	0.824
Blood sample to check HbA1C unknown (ref: withinthe past 12 months)			
PAM level 2	-0.09	0.41	0.823
PAM level 3	0.24	0.37	0.508
PAM level 4	-0.09	0.45	0.850
Blood sample to check cholesterol level over 12 months ago or never (ref: within the past 12 months)			
PAM level 2	0.09	0.29	0.767
PAM level 3	-0.04	0.29	0.896
PAM level 4	-0.09	0.32	0.784
Blood sample to check cholesterol level unknown (ref group: within the past 12 months)			
PAM level 2	-0.68	0.41	0.102
PAM level 3	-0.45	0.37	0.221
PAM level 4	-0.75	0.46	0.107
Blood pressure measurement by healthcare provider over 12 months ago or never (ref: within the past 12 months)			
PAM level 2	-0.77	0.56	0.174
PAM level 3	-0.46	0.49	0.346
PAM level 4	-0.32	0.50	0.524
Blood pressure measurement by healthcare provider unknown (ref: within the past 12 months)			
PAM level 2	-16.00	1822.19	0.993
PAM level 3	-0.23	0.89	0.792
PAM level 4	-15.90	1734.41	0.993
Feet check by doctor over 12 months ago or never (ref: within the past 12 months)			
PAM level 2	-0.44	0.20	**0.025**
PAM level 3	-0.29	0.18	0.105
PAM level 4	-0.58	0.21	**0.005**
Feet check by doctor unknown (ref: within the past 12 months)			
PAM level 2	-0.27	0.64	0.669
PAM level 3	-0.12	0.58	0.838
PAM level 4	-0.83	0.74	0.262
Eye examination over 24 months ago or never (ref: within the past 24 months)			
PAM level 2	-0.54	0.32	0.089
PAM level 3	-0.28	0.28	0.329
PAM level 4	-1.11	0.38	**0.003**
Eye examination unknown (ref: within the past 24 months)			
PAM level 2	0.70	0.76	0.361
PAM level 3	-0.49	0.94	0.605
PAM level 4	-13.75	614.57	0.982
**Self-reported clinical values**			
Level of blood glucose between 7%- 9% (ref: between 2%-7%)			
PAM level 2	-0.28	0.20	0.152
PAM level 3	-0.30	0.19	0.104
PAM level 4	-0.28	0.20	0.161
Level of blood glucose between 9%- 24% (ref: between 2%-7%)			
PAM level 2	-0.44	0.39	0.261
PAM level 3	-0.69	0.40	0.080
PAM level 4	-0.30	0.40	0.447
Level of blood glucose unknown (ref: between 2%-7%)			
PAM level 2	-0.40	0.20	**0.047**
PAM level 3	-0.45	0.19	**0.018**
PAM level 4	-0.75	0.22	**<0.001**
Diastolic blood pressure 90 or higher (ref: lower than 90)			
PAM level 2	-0.27	0.23	0.236
PAM level 3	-0.24	0.22	0.293
PAM level 4	-0.46	0.25	0.061
Diastolic blood pressure unknown (ref: lower than 90)			
PAM level 2	-0.74	0.22	**0.001**
PAM level 3	-0.45	0.20	**0.028**
PAM level 4	-0.99	0.24	**<0.001**
Systolic blood pressure 140 or higher (ref: lower than 140)			
PAM level 2	-0.06	0.18	0.745
PAM level 3	0.07	0.17	0.675
PAM level 4	-0.19	0.18	0.303
Systolic blood pressure unknown (ref: lower than 140)			
PAM level 2	-0.66	0.24	**0.006**
PAM level 3	-0.47	0.22	**0.034**
PAM level 4	-0.72	0.25	**0.004**

Note. Coefficients represent the results of individual linear regressions with PAM level as predictor variable for each health-related outcome while controlling for gender, age, education level, ethnicity, spoken language at home, disease duration and general health status.

Ref = reference group.

Bold values indicate a p-value < 0.05.

## Discussion

This study expands the evidence base on the relationships between patient characteristics, health-related outcomes and patient activation among people with diabetes. The mean patient activation level of respondents was 57.4 on a theoretical scale of 0–100 and resembled that of the general Dutch population (56.9) and of members with diabetes of one’s of American’s largest not-for-profit health plans (i.e., Kaiser Permanente Medical Care program, 57.1) [6, 18]. Other studies reported higher patient activation levels both among people with diabetes and Dutch chronically-ill people [5, 7, 15, 17, 20]. In the current study, patient activation was related to age, education level and self-reported health. Since the participants were relatively old, low educated and often reported a bad health, this might explain the relatively low activation level. However, the mean activation level as measured with the PAM seems to vary across studies between just below 60 up to nearly 70 [5, 6, 11, 12, 15, 18]. This hinders any firm statements as to whether Dutch people with diabetes are relatively inactive when it concerns their healthcare management.

As in other studies, we found that patient activation was related to several patient characteristics [6, 7, 10, 11, 15, 21, 22]. More specifically, men, younger people, higher educated people, those with a better self-reported health and those who spoke Dutch at home reported a higher activation level. We found no association between patient activation and ethnicity or disease duration. One consistent finding across studies is the positive relation between patient activation and self-reported health status. This makes sense given the definition of patient activation; those with a higher activation level report to have more knowledge, skills, confidence and behaviors needed for self-managing one’s condition or health [9, 10]. Longitudinal studies are required to determine the causal direction between patient activation and self-reported health status. It might also be that people feel less in control as their health declines [11].

Previous studies, among diabetes and other patient groups, revealed that patients with a higher activation level were more likely to engage in healthy behaviors such as regular exercise and a proper diet, to engage in disease-specific self-management behaviors, to report medication adherence, and to obtain preventive care [8, 11–15, 23, 24]. As Rask et al., we, however, found no differences in life-style (i.e., smoking) and self-management behaviors (i.e., self-checking blood glucose levels) depending on the patient activation level [15]. Also, we did not find an association between the patient activation level and most of the healthcare utilization measures after controlling for relevant patient characteristics. Only the time since the last feet and eye examination were related to the patient activation level; those with a low activation level (PAM level 1) more often reported they did not have an feet examination in the last 12 or months or an eye examination in the last 24 months. The most obvious result was that those with a high activation level (PAM level 2 to 4) more often reported to have knowledge about diabetes in general and of their own values on clinical indicators compared to people with the lowest activation level (PAM level 1). These results confirm once again the validity of the PAM when it comes to measuring the knowledge needed for self-managing one’s condition or health. Patient activation level as measured with the PAM might therefore especially be predictive of the knowledge people have concerning their health and health care and to a lesser extent of their skills and behaviors to manage their health. Other research likewise showed that those who are more activated are more likely to seek and find health information and to understand it better [11, 23]. Since knowledge about diabetes and clinical values is a prerequisite for patients to be able to control their diabetes, it is important to identify the lower activated patients and explain the meaning of blood glucose levels and how they can control them in a way they can understand.

That we did not find an association between the patient activation level and most healthcare utilization measures, could be explained by differences in the methods used. Like in the present study, Rask et al. also did not find a relationship between the patient activation level and the uptake of routine checkups (except for getting eye examinations) [15]. Both studies based healthcare utilization on self-report of the patient. Using clinical data, Remmers et al. did find a relationship with the uptake of several laboratory tests [6]. Given that clinical data are probably a better indicator of actual healthcare utilization, we recommend to include this kind of data or claims data in future research. Also the type of care seems to be important. We looked at the utilization of routine checkups. Both Remmers et al. (clinical data) and Begum et al. (self-report) included the utilization of hospital care and found that lower activated patients were admitted to the hospital more often [6, 7]. This makes sense given that routine checkups will more often take place on request of the healthcare provider and independent of patient characteristics. The use of unintended healthcare such as a hospital admission will probably be more affected by someone’s activation level. Patients with a lower activation level will have more trouble to properly manage their diabetes which may lead to complications that need treatment in the hospital.

The current study has some limitations. The people with diabetes were sampled and recruited via several health insurance companies. These companies serve the whole population of people with diabetes in the Netherlands and the respondents were selected randomly without excluding any subgroups. However, the fact that people were invited by their health insurer instead of their health care provider may explain the relatively low response. It has been shown that people are more inclined to respond to a questionnaire when it’s sender is more familiar or when one feels connected to them [25]. The low response rate may have introduced certain biases. For instance, men responded more often than women. It also seems plausible that those with a low patient activation level, and thus with a low level of knowledge and skills to manage their health, might be less likely to return the questionnaire. As a result, we might have underestimated the relations between patient activation level and the health-related outcomes.

The study was cross-sectional impeding any conclusions on the causal directionality between patient activation level and health-related outcomes. So far, only two studies used a longitudinal design and showed that an increase in patient activation is associated with more self-management behaviors and that a higher patient activation level results in better health-related outcomes two years later [6, 14]. These studies thus suggest that increasing patient activation will improve health-related behavior and outcomes.

Finally, the clinical indicators were based on self-report and only concerned the last measurement by a healthcare provider. Therefore, we could not determine whether a higher patient activation level was associated with better diabetes control or better physical health. The results did show that the recall of values on clinical indicators was positively related to patient activation; an interesting finding on its own. Comparable trends were found for the healthcare utilization measures but here there was a problem with statistical power. Far out most people reported to have received checkups according to the Dutch national guidelines. Only a small group of patients could not remember when their last checkups had been and those patients appeared to score substantially lower on patient activation.

## Conclusions

People with diabetes are more and more expected to be active patients, that is to be in charge of their own health and the healthcare they receive. One way to accomplish this is by educating and training people to get involved, be well-informed and able to adjust their behavior to maintain a good health. We reinforced prior findings among a sample of Dutch people with diabetes and showed that patient activation differs between patient subgroups. The study also yielded that patient activation level as measured with the PAM was especially related with disease-specific knowledge and less with health-related behavior, healthcare use and self-reported clinical values. We provided several explanations why the expected associations with health-related behavior and healthcare use were not found in the present study.

The results implicate that the PAM might be an useful instrument for clinical practice to identify people with diabetes most in need for diabetes education. The PAM is a short self-report questionnaire that can easily be implemented in regular diabetes care. The next step is to implement and evaluate the effectiveness of interventions to increase patient activation or to tailor diabetes care to someone’s activation level. The first results on such interventions are promising [8, 13, 14].
